# A framework to explore micronutrient deficiency in maternal and child health in Malawi, Southern Africa

**DOI:** 10.1186/1476-069X-8-S1-S13

**Published:** 2009-12-21

**Authors:** Natalie Dickinson, John Gulliver, Gordon MacPherson, John Atkinson, Jean Rankin, Maria Cummings, Zoe Nisbet, Andrew Hursthouse, Avril Taylor, Chris Robertson, Wolfgang Burghardt

**Affiliations:** 1 School of Health Nursing & Midwifery University of the West of Scotland, Paisley, PA1 2BE, UK; 2 School of Engineering and Science, Unviversity of the West of Scotland, Paisley, PA1 2BE, UK; 3 School of Social Sciences, University of the West of Scotland, Paisley, PA1 2BE, UK; 4 Department of Statistics & Modelling Science, University of Strathclyde, Glasgow G1 1HX, UK; 5 Soil Technology, Department of Biology & Geography University Duisburg-Essen, 457 Essen, Germany

## Abstract

**Background:**

Global food insecurity is associated with micronutrient deficiencies and it has been suggested that 4.5 billion people world-wide are affected by deficiencies in iron, vitamin A and iodine. Zinc has also been identified to be of increasing concern. The most vulnerable are young children and women of childbearing age. A pilot study has been carried out in Southern Malawi, to attempt to link the geochemical and agricultural basis of micronutrient supply through spatial variability to maternal health and associated cultural and social aspects of nutrition. The aim is to establish the opportunity for concerted action to deliver step change improvements in the nutrition of developing countries.

**Results:**

Field work undertaken in August 2007 and July/August 2008 involved the collection of blood, soil and crop samples, and questionnaires from ~100 pregnant women. Complex permissions and authorisation protocols were identified and found to be as much part of the cultural and social context of the work as the complexity of the interdisciplinary project. These issues are catalogued and discussed. A preliminary spatial evaluation is presented linking soil quality and food production to nutritional health. It also considers behavioural and cultural attitudes of women and children in two regions of southern Malawi, (the Shire Valley and Shire Highlands plateau). Differences in agricultural practice and widely varying soil quality (e.g. pH organic matter, C/N and metal content) were observed for both regions and full chemical analysis of soil and food is underway. Early assessment of blood data suggests major differences in health and nutritional status between the two regions. Differences in food availability and type and observations of life style are being evaluated through questionnaire analysis.

**Conclusion:**

The particular emphasis of the study is on the interdisciplinary opportunities and the barriers to progress in development support in subsistence communities. Engaging at the community level and the balance of expectations from both study subjects and research team highlight the merit of careful and detailed planning and project delivery.

## Introduction

Micronutrient malnutrition is a national health problem in Malawi, a small, land-locked sub-Saharan African country [1-4]. In many developing countries numerous agricultural as well as social factors are constraints that contribute to food insecurity and malnutrition [5]. A review of the context for Malawi [6] highlights the scope for interdisciplinary research to investigate the acknowledged gap between knowledge and successful implementation of interventions to address nutritional inadequacy. This pilot project established a protocol for research in Malawi, incorporating bottom-up participatory approaches through the construction of a dynamic and engaged interdisciplinary research team.

## Developing a methodology

Ethical permission for this study was granted both by the University of Paisley (now UWS); Research Ethics Advisory Group and the College of Medicine, University of Malawi; Research Ethics Committee (COMREC). All participants were recruited after extensive sensitisation activities and signed agreement to participate.

The study targeted pregnant women (~100) from two geographically different regions close to Blantyre in S. Malawi, where diet is determined by home grown food (dominated by maize). Samples of blood, crop and soil were collected for analysis and a lifestyle questionnaire completed. Many confounding factors are recognised but it was considered that focusing efforts to collect evidence across all components would provide important observational data and understanding in the two regions:

a. The Shire Highlands, a plateau region to the east of the Shire River (*Chiradzulu *district). Relatively densely populated, good agricultural productivity, 900-1100 m elevation, experiencing relatively cool temperatures and regional average rainfall [7,8].

b. The low-lying Shire Valley (*Chikwawa *district), ~100 m elevation, higher average temperatures and lower rainfall [7], although the area floods every year and farmers struggle to produce enough food to last the year [9].

**Figure 1:**
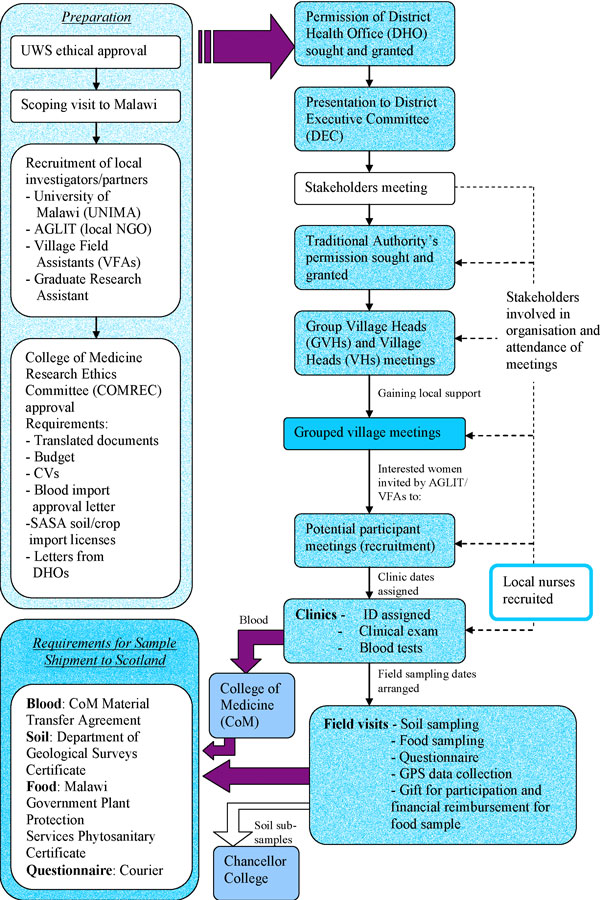
**Multidisciplinary maternal health research in Malawi - a schematic diagram of project development highlighting stakeholder permissions and authorisation**.

Local ethics committee approval involved gaining permission at multiple levels of government and rural authorities, and 'sensitization' of the communities who would be involved (important for new or unusual actions). Sensitisation messages provide essential information to the community about what is being done, why, when and by whom, and who will be involved [10]. It follows traditional hierarchy of the local community and is necessary for communities to stand the best chance of accepting interventions and involvement from outside, providing the opportunity for research teams to disseminate project aims and collect preliminary feedback. The process is shown as part of Figure 1, which reveals the extensive interactions required. A locally based team was built working with AGLIT (a local NGO in Chikwawa), two Village Field Assistants (VFAs) in Chiradzulu, and a locally trained Malawian Research Assistant (RA). A UK member of the team was resident in Malawi, providing logistical support, recording dialogue within the research group and observational data on human behaviour and government policy issues relevant to the project. This included findings concerning agriculture, eating habits, family life, and health care.

The women recruited to participate had all sought permission from their husbands and had signed consent forms.

### Blood sampling and clinics

Blood sampling clinics were run over two weeks and the height, weight and Estimated Delivery Date (EDD) of each participant was recorded within the questionnaire along with participant identification (ID) number.

Venepuncture (blood-taking) and clinical examination (including fundal height, a measurement to approximate the stage of pregnancy) were carried out by local nurses hired to assist in the study, and supervised by a nurse or midwife from the project team. Approximately 15 ml of blood was drawn and decanted into three specific blood bottles and split for analysis in Malawi and the UK.

### Field visits - soil, food and questionnaires

Soil sampling, food collection and lifestyle questionnaire were undertaken simultaneously. Each participant was visited at her home; a GPS location was recorded at the participant's house, and photographs were taken.

a. Soil: samples were collected following local governmental procedures [11,12]. Composite samples (~25 points per hectare, hand auger) from: Chiradzulu (ridge/furrow management) 0-10 cm and 10-25 cm on ridges; 0-10 cm in between rows; Chikwawa (un-furrowed) 0-10 cm and 10-25 cm. Field description included GPS readings of field outline and centre as well basic landscape observation. A subset of soils was provided for local chemical analysis (Chancellor College, University of Malawi, Zomba) and samples packaged for shipment to the UK.

b. Crops: food samples from the previous harvest were stored at the participants' house. At each home, their food store was photographed and a small (handful) sample collected. A donation was offered to each participant for the food samples taken and samples packaged for shipment to the UK.

c. Questionnaires: The questionnaires were created to assess health data which may be relevant to the understanding of linkages of the micronutrient content of the soil, food and blood samples. The questions related to the current pregnancy, dietary assessment, and immediate environment which may impact on health. Due to low levels of literacy amongst the study population, the questionnaire was administered in private by a native Chichewa-speaking interviewer - to achieve a higher response rate to questions, and increase reliability of the questionnaire.

## Results and discussion

### Creation of a comprehensive, interdisciplinary methodology for research in Malawi

Successful execution of the project required an understanding of and engagement with the local population. Key observations were:

a. Main academic contact: a partnership with the University of Malawi (UNIMA), College of Medicine (Blantyre), (following project authorisation from the Secretary of Foreign Affairs, Malawi Government). Ethical approval from the College of Medicine Research Ethics Committee (COMREC) formally identified a local academic contact and required that Malawian laboratories and researchers would be used where possible. Much of the infrastructure available was unsuited to study requirements. Approval was only reached after a lengthy cycle of iteration of COMREC applications.

b. Engaging a local NGO: Despite AGLIT's previous experience of this type of study, the detailed negotiation of roles and responsibilities and differences in priorities and views of project ownership created a major delay in delivery until budgets had been negotiated. AGLIT undertook the process of gaining permissions and working through the rural hierarchy down to village level.

c. Local independent researchers (RA/VFA): all had excellent local knowledge and were independent of local political or financial agenda with very strong personal motives to help improve lives locally.

d. Engaging community stakeholders: Resistance was met only at the District Executive Committee meetings due to the study being academic rather than a charitable venture. The stakeholder meetings ensured the smooth running of the project, however some stakeholders appeared to be involved more for the financial incentive. Important stakeholders were the police (present at all village level meetings, due to the sensitive nature of blood-taking), and the staff of the District Health Office. Approval from the Traditional Authorities (and Village Heads) was essential in order to gain access to the study population. No resistance was met at this level, many questions were asked, and the rural leaders were keen that results of the study should be fed back to the communities, so that they may gain something from taking part. Sensitisation meetings went well where information was presented and questions were answered. Recruiting women to take part in the study was not a problem.

e. Fieldwork/Sampling: Sampling was broken down into two phases. Blood samples were taken at specially arranged clinics as it was not culturally acceptable (or safe for the research team) to take blood outside a clinical setting. The clinics worked well, and the coding system developed ensured that the participants were all easily identifiable. Soil/crop collection was straightforward.

f. Sample receipt and treatment: import to the UK of soil/food required authorisation and protocol for materials handling and disposal and verification in Malawi. Additional authorisation and verification for soil export came from the Department of Geological Surveys (Ministry of Mines, Energy and Natural Resources), and was fast-tracked through partnership with UNIMA. Exporting the blood samples was more simple in terms of documentation (a Material Transfer Agreement from the College of Medicine), but met difficulty in organizing a courier who had the capacity to transfer biological samples.

### Establishing a research team

It is acknowledged that the close involvement of the local community in planning and implementation is essential for successful interventions [13,14]. This was confirmed in Malawi. A range of activities are recommended to help external groups understand affected communities, build relationships with them, and foster participation [10]. This is a two way process, as the communities involved also learn more about the researchers. Key people were identified, the VFAs, who could pass on messages about the study to the rest of the community, and identify and liaise with suitable participants; an integral part of community mobilisation. The use of VFAs in Chiradzulu was found to be a much more effective and successful than the alternative method used in Chikwawa. The endorsement by locally respected and trusted community members increased the interest and participation of the communities.

The recruitment of a local female RA increased the level of trust from the participants in the study. With a similar village background, the RA was able to empathise and communicate easily with the communities, whilst also being able to understand the academic context, and provide advice on the best way to implement the study in rural Malawi.

The relationship with the UNIMA was invaluable with bureaucracy reduced, a number of logistical issues were resolved more easily, cheaply and quickly by being able to identify ourselves as a UNIMA research team, rather than a UK team.

### Initial findings

The project was successful in being able to obtain spatially identifiable samples of soil (~240), food (~250) and blood from 97 individuals. Chemical data will be incorporated within a spatial and epidemiological assessment. Two months of intense field work could only be undertaken after nearly one year of on the ground activity.

Soil and Crops: Emerging data on Fe and Zn levels in the soils confirms field observations of the deeply weathered ferralitic plateau soils and relatively young fluvial deposits in the valley (e.g. 4.1% w/w Fe plateau (n = 40); 2.5% w/w Fe valley (n = 10). Zinc levels are similar in both regions (e.g. 53.5 mg/kg plateau (n = 40), 55.4 mg/kg valley (n = 10). Major soil properties (e.g. pH, C/N) are significantly different between the two regions. Crop samples confirm maize dominance but with supplements of pea/bean varieties and some root crops. Iron and zinc contents are very low ~20 mg/kg for both elements.

**Figure 2:**
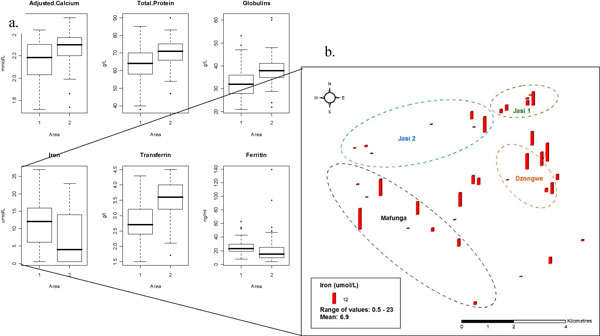
**a. Box plots of selected blood measurements in among the women in two areas of Malawi (area 1 = Chiradzulu, plateau district; area 2 = Chikwawa, river valley); b. Spatial distribution of blood data - selection from villages in area 2**.

Blood: Usable blood was available for 92 women, 46 in each region. Preliminary, unadjusted analyses (see Figure 2), showed significant differences (Wilcoxon test, Bonferroni adjustment) between women in the two areas. Levels of iron deficiency, anaemia, infection and malaria are all higher in Chikwawa (the flood plain), than on the plateau. calcium, protein and globulins were higher in Area 2 (Chikwawa) while iron and ferritin were higher in Area 1 (Chiradzulu) (and consequently transferrin lower and transferrin saturation higher).

Spatial variation: initial evaluation of questionnaires confirms life style variation between locations. Simple measures of distance to fields from the house emphasises large differences in effort required to work the land (e.g. 0.1-1 km on the plateau and 0.5-5.5 km in the valley). Soil management and animal husbandry vary, with less apparent control on both in the valley.

## Conclusion

Successful completion is highly dependent on clear and careful mapping of roles and responsibilities of research teams. Recruitment and retention of reliable local field researchers and recognition of complex permissions and layers of authority must be undertaken to secure willing participation in rural communities.

This has far reaching consequences for the quality of physical and observational data. There are no "magic bullets" to resolve issues surrounding project delivery under these conditions, except engagement at all levels and the persistence of the research team.

A consequence of careful engagement and project execution is the recognition that this work has raised local expectation and interest, with a need to follow up. It offers considerable potential to address questions of micronutrient deficiency, linked to community development.

## Note 

The peer review of this article can be found in Additional file 1.

## Competing interests

The authors declare that they have no competing interests.

## Authors' contributions

ND participated in fieldwork and organisation of permissions and approval and drafting the paper, JG undertook spatial analysis and development of statistical interpretation and drafting of appropriate manuscript sections, GMacP was involved in initial project conception, design, participated in fieldwork and interpretation of blood data and drafting of the manuscript, JA participated in the design of the project, provided logistics and organisational support for fieldwork and in interpreting data and drafting of the manuscript; JR was involved in preparation of questionnaire, field work and sample collection (particularly blood) and drafting of manuscript; MC was involved in preparation of questionnaire, field work and sample collection (particularly blood) and drafting of manuscript, ZN was involved in the fieldwork and organisation of permissions and approval and drafting the paper; AH was involved in initial project conception, design, fieldwork, overseeing and interpreting soil and crop analysis and in the drafting of the manuscript; AT was involved in the initial design of the project, construction of questionnaires and interpretation of the data and drafting of the manuscript; CR was involved in the design of the project, statistical interpretation of data and drafting of the manuscript; WB was involved in the collection of soil and crop samples, soil analysis and interpretation and in the drafting of the manuscript. All authors read and approved the final manuscript.

## Supplementary Material

Additional file 1 Peer review. Click here for file
