# Supplementary material for: A framework to explore micronutrient deficiency in maternal and child health in Malawi, Southern Africa
Source: Environ Health. 2009 Dec 21;8(Suppl 1):S13. doi: 10.1186/1476-069X-8-S1-S13 (PMC2796491; doi:10.1186/1476-069X-8-S1-S13)
Supplement: Additional file 1 — Peer review. [file 1476-069X-8-S1-S13-S1.doc]

| Article ref no. | A1471-2105X SZ-S13 |
| --- | --- |
| Title | **Micronutrient deficiency in maternity and child health: exploring agricultural, medical and social influences on Fe and Zn deficiencies** |
| Author(s) | N Dickinson, J Gulliver et al. |

**Ref#1**

**General comments:**

This is an unusual paper in the sense that it does not follow the normal format for scientific papers. It describes the setting up of the project and the associated problems but gives very little detail on what was done and very few results (just a few examples). The title suggests that the reader is going to read in detail about micronutrient deficiencies in maternity and child care but in fact most of the paper is concerned with the setting up methodology for the project.

**RESPONSE:** Yes, the paper is unusual – we felt the detail of methodology was as important as some of the outcomes of primary data. This will come as a fuller paper after further analysis. We have modified the title to better reflect the aim of the paper. The descriptive nature of the material required considerable shortening to fit the limits imposed; as a result detail is missing.

**Major compulsory revisions:**

The title should better reflect what the paper contains and should include somewhere the words ‘in Malawi’

RESPONSE: Agreed and completed

.

**Minor essential revisions:**

The Materials and Method section has very little detail. For e.g. it describes samples of blood, soil and food being taken but does not say what determinations were carried out on them

Some minor editorial changes:

p. 6 line 21: ‘………from their husbands’ and….’ should be

‘………from their husbands and….’

p. 7 line 3: ‘……..participant recorded………’	should be

‘……..participant was recorded………’

p. 10 line 1: ‘….Blood samples at…’	should be

‘….Blood samples were taken at…’

p. 12 lines 5 and 6: references to Protein, Globulins, Iron, Ferritin and Transferrin should not be capitalized.

RESPONSE: Agreed and completed

**Discretionary revisions:**

Although only preliminary data are given it would have been helpful to have some numerical food chain nutrition linkages. E.g. soils of x Fe content produced crops of y Fe content contributing to a daily Fe intake of y. This was associated with an Fe status of whatever. This is inferred from differences between the regions but a bit more numerical data would add a lot.

RESPONSE: Agreed, partial numerical data added to expand the description of findings. Main aim of paper was to illustrate “process” of engaging at the community level.

**Ref#2**

**General comments:**

Much of the emphasis in this paper is on methodology and setting up the project. While it is appreciated that the project is logistically complex it would benefit from more detail of scientific findings.

RESPONSE: We found the methodology developed for the implementation of this study to be an important outcome of the work. A descriptive paper outlining this was therefore a useful (missing) contribution to the literature.

**Major compulsory revisions:**

More details of analytical methods, data and discussion of data needs to be included before the paper is acceptable for publication.

RESPONSE: More analytical data has been included to reinforce discussions. However, the full analytical description was not added (it would expand the article well beyond the allowed length). The change in title perhaps reflects better the aim of the work and what is included on the methodology, something we find lacking in the literature.

**Minor essential revisions:**

Malawi should be included in the title.

RESPONSE: Completed
